# Linear Control Theory for Gene Network Modeling

**DOI:** 10.1371/journal.pone.0012785

**Published:** 2010-09-16

**Authors:** Yong-Jun Shin, Leonidas Bleris

**Affiliations:** 1 Department of Electrical Engineering, University of Texas at Dallas, Richardson, Texas, United States of America; 2 Department of Bioengineering, University of Texas at Dallas, Richardson, Texas, United States of America; Fondazione Telethon, Italy

## Abstract

Systems biology is an interdisciplinary field that aims at understanding complex interactions in cells. Here we demonstrate that linear control theory can provide valuable insight and practical tools for the characterization of complex biological networks. We provide the foundation for such analyses through the study of several case studies including cascade and parallel forms, feedback and feedforward loops. We reproduce experimental results and provide rational analysis of the observed behavior. We demonstrate that methods such as the transfer function (frequency domain) and linear state-space (time domain) can be used to predict reliably the properties and transient behavior of complex network topologies and point to specific design strategies for synthetic networks.

## Introduction

Cells comprise of multiple, heterogeneous subunits that operate in a well-orchestrated manner [1], [2]. Although extremely complex, phenotypes such as cell division and environmental adaptation are the outcome of discrete changes that lead to a deterministic sequence of information transfer and processing within the cell. This information is encoded and transferred via multiple pathways, in different time-scales, and is typically processed in parallel by multi-component networks. Such networks comprise of genes, gene products and small molecules that mutually affect each other or interconvert through biochemical reactions. The number of possible network topologies for a given set of elements is large and it grows exponentially with the number of elements.

Systems biology is an interdisciplinary field that aims at understanding such complex interactions in cells, via the use of a wide spectrum of theoretical and experimental techniques [3]. One of the main thrusts of systems biology is the study of gene networks [4]–[7], via top-down and bottom-up approaches [8]. A top-down approach aims at unraveling the complexity of network dynamics without or with little prior knowledge of the network components and the relationships between them [9]–[12]. On the other hand, a bottom-up approach (closely related to synthetic biology) aims at constructing and studying small-scale biological networks from modular components [13]–[17]. Gene networks are inherently stochastic [18], [19], which renders any modeling effort nontrivial. Furthermore, as the size of a network increases, it becomes increasingly difficult to predict its dynamic behavior. Another characteristic feature is the existence of nonlinearities in biological networks, which further complicates any modeling effort.

Towards analyzing the dynamic behavior of gene networks, a range of mathematical and computational modeling methods have been developed, including Boolean networks, Petri nets, state-charts, ordinary differential equations, and stochastic simulation algorithms [4], [20]–[30]. These approaches can be further organized into two larger categories: logical and continuous models. Logical models deal with the logical sequence of events while continuous models describe the dynamics that depend on molecular concentrations and time.

In this paper, we develop and use linear models [31]–[34], which are shown to capture the dynamics of gene networks in an intuitive and efficient way. We argue that tools of linear control theory, including transfer function (frequency domain) and linear state-space (time domain) methods can be exceptionally practical for systems and synthetic biologists towards unraveling the properties of gene networks and engineering novel systems. We provide several examples of the application of the transfer function method for the analysis of gene networks, starting with network motifs [5], the basic building blocks of gene networks. The transfer function method is sometimes regarded as “classical” in control theory. The state-space or “modern” approach describes a system as a set of input, output, and state variables related by first-order differential or difference equations. One of the advantages of using the state-space method is that it can be used to model multiple-input multiple-output (MIMO) gene networks in a compact manner, utilizing vectors and matrices. Furthermore, we show that the linear state-space approach enables us to utilize a spectrum of tools available for optimal/robust estimation/control for gene network modeling [35]–[37]. As an example, we illustrate that the Kalman filter, one of the well-established optimal estimation tools, can be applied for stochastic modeling of a simple two-gene network. Finally, using a six-node gene network, we demonstrate that our linear approaches can reduce the modeling complexity and provide rapid insight about its dynamic behavior, as compared to conventional non-linear modeling approaches.

The manuscript is organized as follows. We commence our analysis with modeling a simple gene regulation case and subsequently provide the details behind the proposed linearization scheme. Using these results we present the transfer function method for gene network modeling and exemplify the methodology for collapsing cascade and parallel forms to transfer functions. We then provide six case studies that cover a wide range of systems and synthetic biology problems including: cascaded simple regulations, synthetic gene oscillators, effect of the basal production rate, four cascaded simple regulation loops, and finally interconnected feedforward loops. Subsequently, we present the linear state-space method for gene network modeling and we outline its use for the analysis of cascaded simple regulations. Using the linear state-space formulation, we conclude this manuscript with two additional case studies: the use of optimal estimation in gene network measurements and the analysis of the six-node gene network.

### A. Modeling simple gene regulation

**Figure 1:**
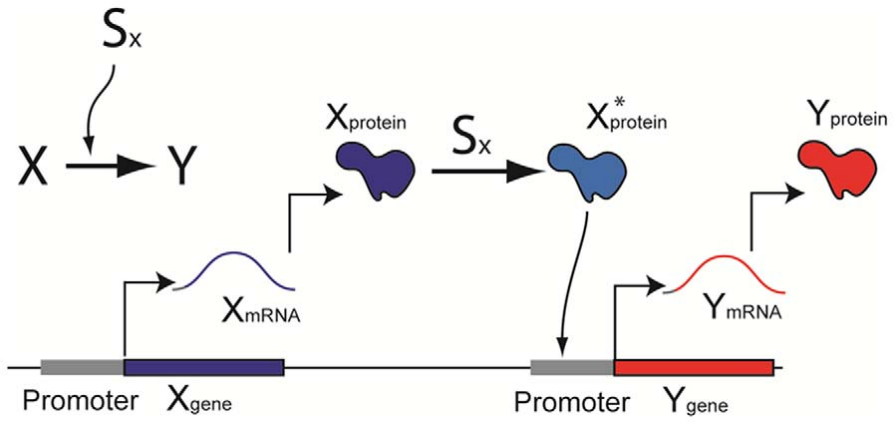
A schematic illustration of simple gene regulation.

A two-gene network (or simple regulation) can be regarded as a fundamental unit that serves as a basic building block for constructing elaborate networks. In simple regulation, one gene (*Y_gene_*) can be activated by another gene (*X_gene_*), as indicated by the notation X→Y in **Figure 1**. This notation, however, involves in reality multiple steps. First, *X_gene_* is transcribed into a messenger RNA, *X_mRNA_*, which is then translated into a protein (*X_protein_*). In the presence of a signal *S_x_*, *X_protein_* shifts to its active form X*_protein_ (also called a transcription factor of *Y_gene_*) and binds the promoter of *Y_gene_*, transcribing *Y_gene_* into *Y_mRNA_*. Finally, as *Y_mRNA_* is translated, *Y_protein_* is produced. The signal *S_x_* acts like a switch, by determining the amount of active form *X_protein_* or X*_protein_. The production rate of *Y_protein_* can be expressed as a function of time *F*(*t*) (units of concentration per unit time). The production is balanced by processes that decrease *Y_protein_*, namely degradation (protein destruction) and dilution (concentration reduction due to the increase of cell volume) [13]. Degradation and dilution can be collectively denoted as a time-dependent function *d(t)*. The change in the concentration of *Y_protein_* depends on both *F(t)* and *d(t)*, and its dynamics can be described as an ordinary differential equation:
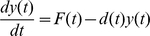
(1)where *y*(*t*) stands for the concentration of *Y_protein_*. If we assume that *d*(*t*) depends only on dilution (for example, in growing bacteria, many proteins are stable and they are not actively degraded), *d*(*t*) can be expressed as a constant *D*, based on following equation [13]:
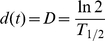
(2)where *T*_1/2_ is the response time, the time to reach one half of the steady-state *Y_protein_* concentration. The response times of bacteria are approximately 30 min to a few hours, while those of eukaryotic cells can be longer [13]. Equation 1 can now be restated by substituting *d(t)* by the constant *D*:
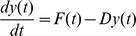
(3)


For the analysis of the function *F(t)*, *w*e need to consider a number of additional factors. As stated earlier, *X_protein_* must be converted to X*_protein_ by the signal *S_x_* in order to initiate the *Y_protein_* production. The concentration of X*_protein_ can be expressed as a function of *S_x_*, which is an activating switch. This switch-like relationship can be described using the non-linear Hill function [13], and the relationship between X*_protein_ (we use x*(*t*)) and *S_x_* (we use *s_x_*(*t*)) can be expressed as:
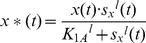
(4)where *x*(*t*) stands for the concentration of total *X_protein_* that includes both inactive and active forms. It is the maximal level of X*_protein_ or x*(*t*) (in units of concentration) that is reached when *s_x_*(*t*)≫*K_1A_*. *K_1A_* is the concentration of *s_x_*(*t*), at which half-maximal concentration of x*(t) is reached. The Hill coefficient *l* determines the steepness of the function. Note that *K_1A_* and *l* are determined by a number of factors, such as enzymatic activity, pH, and temperature, and can be estimated from experimental data. When *s_x_*(*t*) acts as a repressor, the corresponding Hill function is :
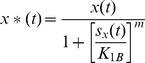
(5)where *x*(*t*) is the maximal level of the x*(*t*) production that is reached when *s_x_*(*t*) = 0. In other words, *x*(*t*) is reached (x*(*t*) = *x*(*t*)) when there is no signal causing repression. *K_1B_* is the concentration of *s_x_*(*t*), at which half-maximal repression of the x*(*t*) production is reached. Again, *m* is the Hill coefficient and determines the steepness of the function.

As a generalization, we can assume that *F*(*t*) is the sum of a basal promoter production rate *F_0_* and the Hill function:
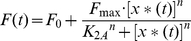
(6)
*F_max_* is the maximal level of the *Y_protein_* production (in units of concentration per unit time) that is reached when x*(*t*)≫*K_2A_*. *K_2A_* is the concentration of x*(*t*) at which half-maximal production of *Y_protein_* is reached. *n* is the Hill coefficient, and *K_2A_* and n are determined by a number of factors such as the translation rate, pH, and temperature. Similarly, when x*(*t*) acts as a repressor, the corresponding Hill function is:
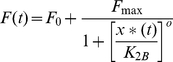
(7)where *F_max_* is the maximal level of the *Y_protein_* production that is reached when x*(t) = 0. *F_max_* is reached when there is no transcription factor causing repression. *K_2B_* is the concentration of x*(t) at which half-maximal repression of the *Y_protein_* production is reached. *o* is the Hill coefficient.

### B. Linearization

**Figure 2:**
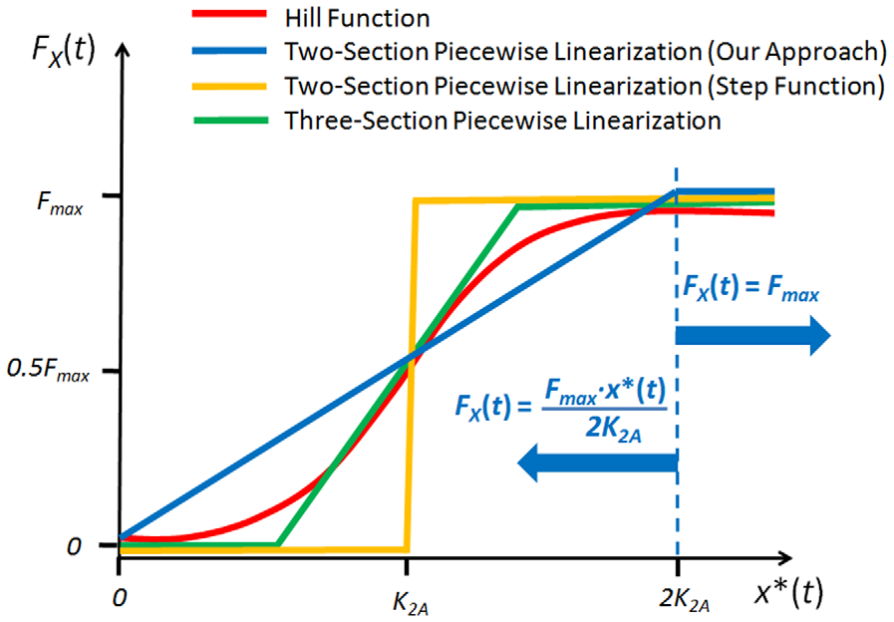
Linearization of the relationship between *F*(*t*) and x*(*t*). The red line represents the Hill function. The linear approximation is represented by a blue line in two distinct regions separated by a dotted line. The logic approximation (step function) is shown in yellow and the three-section piecewise linearization is shown in green.

A non-linear model can be linearized around an equilibrium point using a Jacobian matrix, providing insight about the network behavior near that point [38]. For systems that involve Hill functions, there have been various linearization approaches that can be used to study the dynamics outside of the equilibrium [13], [39]–[41]. These methods enable us to convert a non-linear model into a linear model and use linear tools for studying network dynamics over a wide range of parameter and variable values. Two such approaches widely discussed in the literature [13], [39]–[41] are the two-section piecewise linearization (step function or logic approximation) and the three-section piecewise linearization (**Figure 2**). In the figure, *F_x_*(*t*) represents the production rate of *Y_protein_* due to *X_protein_*. In this section we introduce an alternative linearization approach and in later sections we will illustrate how the particular approach enables us to capture the dynamics of gene networks in an intuitive and efficient way.

Our method is a variation of a two-section piecewise linearization. In the step function approach (yellow in **Figure 2**), *F_x_*(*t*) is zero for all the values of x*(*t*) between 0 and a threshold point (TP) and takes its maximum rate when x*(*t*) is above TP. Therefore, the step function is inherently less accurate than the Hill Function and results to loss of information. When using the three-section piecewise linearization approach (green in **Figure 2**) one has to identify the two threshold values. As shown in **Figure 2**, using our approach we are required to identify one threshold. Furthermore, we may set our threshold higher than *2K_2A_*, the x*(*t*) value required for reaching *F_max_*, thus eliminating the need for explicitly incorporating the threshold into our model.

The first step towards applying linear control theory tools is to reduce (3) to a linear equation, in other words express *dy*(*t*)/*dt* as a linear combination of x(t) and y(t). The concentration x*(*t*) of (4) can be expressed as the product of *x*(*t*) and a function *F_1_*(*t*) that depends only on *s_x_*(*t*) as shown in (8). Note that *l* and *K_1A_* are constant values determined experimentally, and *F_1_*(*t*) is a scaling factor that always lies between 0 and 1, depending on the value of *s_x_*(*t*). When *s_x_*(*t*) is zero, *F_1_*(*t*) is also zero, and if *s_x_*(*t*) is very large, then *F_1_*(*t*) approaches one.
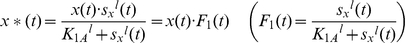
(8)


The Hill function relationship between *F_x_*(*t*) and x*(*t*) in (6) can be plotted as shown in **Figure 2** (red line). As described earlier, *F_max_* is the maximal level of the *Y_protein_* production that is reached when x*(t)≫*K_2A_*, and *K_2A_* is the concentration of x*(*t*) at which half-maximal production of *Y_protein_* is reached.

For linearization, we will consider two cases: a case when x*(*t*) is smaller than *2K_2A_* and another when it is greater than *2K_2A_* (**Figure 2**). For the first case, we will assume that a line that passes through the origin (0,0) and (*2K_2A_*, *F_max_*) represents the linear approximation of the Hill function, and the slope of the line is:
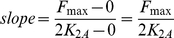
(9)Now, denoting the slope as *F*_2_, (6) can be expressed as:
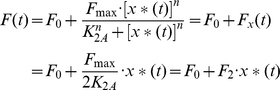
(10)Substituting x*(*t*) in (10) by *x*(*t*)·*F*_1_(*t*) from (8), (10) becomes:
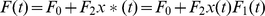
(11)Denoting *F*_2_·*F*_1_(*t*) as *f*(t), (11) becomes:
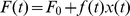
(12)Substituting *F*(*t*) by *f*(*t*)*x*(*t*), (1) can now be expressed as:
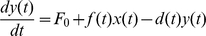
(13)where dy(t)/dt is shown as a linear combination of x(t) and y(t).

For the x*(t) values greater than *2K_2A_*, *F(t)* is equal to the constant *F_max_* (**Figure 2**). Therefore, (1) can be expressed as:
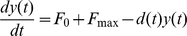
(14)which again shows that dy(t)/dt is a linear combination of *F_max_* and y(t). (13) and (14) can be shown together as:
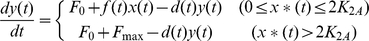
(15)Similarly, for the case when *s_x_*(*t*) acts as a repressor as shown in (5), x*(*t*) can be expressed as :
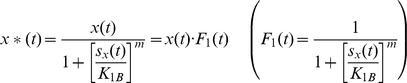
(16)When x*(t) acts as a repressor, based on the linearization scheme shown in (9–12), the corresponding Hill function (7) can be expressed as:
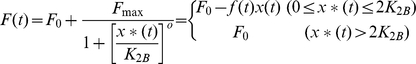
(17)From now on, we will consider only the non-trivial cases when x*(t) is between zero and 2*K_2A_*.

## Results and Discussion

### A. Transfer function method for gene network modeling

A transfer function can be derived from a linear, time-invariant differential equation using Laplace transform [42]. It is a mathematical representation of the relationship between the input and output in the frequency domain. Towards analyzing a gene network, the transfer function method can provide useful information about the behavior and stability of a system. Furthermore, using block diagrams, the transfer function method can represent large complex structures in a simple and intuitive way. Notably, the stochastic nature of gene networks has also been analyzed in the frequency domain [43]–[45].

#### 1. Simple regulation

**Figure 3:**
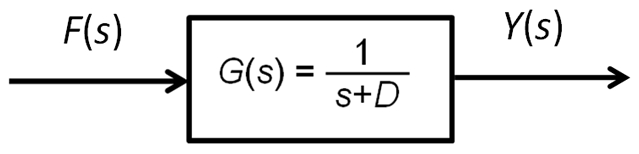
Block diagram representation of simple regulation.

Starting from equation (8), if we assume that the concentration of the signal *s_x_*(*t*) is constant or time-invariant (for example, being at saturation), then we can assume *F*_1_(*t*) is also a constant value, *F*_1_. Now (3) can be rewritten as:
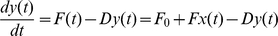
(18)This time-invariance is required for the application of the transfer function method [42]. Using the Laplace transform, (18) can be expressed as:
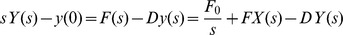
(19)where *F(s)*, *X(s)*, and *Y(s)* represent the Laplace transform of *F*(*t*), *x*(*t*), and *y*(*t*). Once the Laplace transform is evaluated, computing in Laplace domain is algebraic and the complexity of solving differential equation is eliminated [42] [41] [41] [42] [43] [48]. Assuming the initial concentration of *Y_protein_*, *y*(0), is zero, the transfer function *G*(*s*), which relates the input *F*(*s*) and output *Y*(*s*) in the frequency domain, can be expressed as:
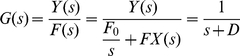
(20)Note that the constant *D* plays a critical role in *G*(*s*). It implies that the critical factor that characterizes this system is the dilution/degradation constant. Also in this case the transfer function *G*(*s*) is not affected by the input *F_0_*. However, as the network becomes more complicated (e.g., with positive and negative feedbacks), *F*_0_/*s* is incorporated into the transfer function. The block diagram representation of the input, output, and transfer function of simple regulation is shown in **Figure 3**. Using the inverse Laplace transform, the output in the time domain, *y(t)*, can be calculated as following :
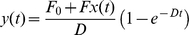
(21)In case *x(t)* is not changing (has reached its steady state) and set as a constant value *X*, *F·x(t)* becomes *FX* and the resulting step response is:
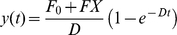
(22)where *y(t)* will approach the steady state value as time *t* goes to infinity, and the constant *D* in *e^−Dt^* determines how fast the steady state is reached. The step response in Laplace domain can be expressed as:
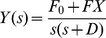
(23)


Solving a differential equation or evaluating the inverse Laplace transform enables us to evaluate the output response of a system. However, these techniques can be laborious and time-consuming. The use of poles and zeros, fundamental to the analysis and design of control systems, is a technique that can simplify the evaluation. Given the first-order transfer function *G(s)* in (20), a pole, the value for *s* which makes the denominator of the transfer function equal to zero, exists at *s* = −*D*. Since *D* (the degradation/dilution factor) is greater than zero in a real biological system, −*D* can be assumed to be a negative number. This indicates that the system is stable because stable systems do not have any positive poles [42]. For a system to be stable, its natural response must decay to zero, as time approaches infinity, or oscillate. There are various specifications (time constant, rise time, and settling time, etc) that may reveal useful information about the network behavior (the reader is referred to [42]). 1/*D*, called the time constant of the response, is the time for the step response to rise 63% of its final or steady state value. Rise time, defined as the time for the system response to go from 0.1 to 0.9 of its steady state value, is equal to 2.2/*D*. Settling time is the time for the response to reach and stay within 2% of its final value and it is equivalent to 4/*D*.

#### 2. Cascade and parallel forms

**Figure 4:**
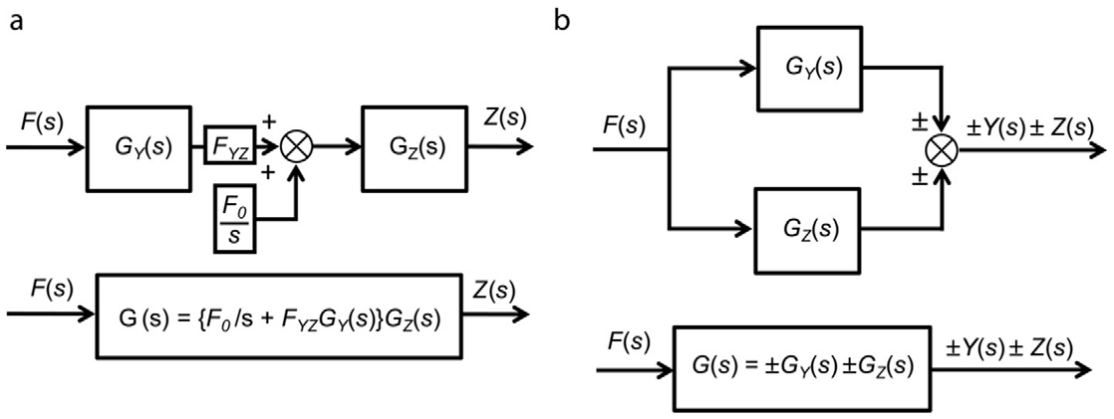
Cascade and Parallel forms. (a) Two cascaded simple regulations (above) and their equivalent simplified form (below). (b) Parallel simple regulations (above) and their equivalent simplified form (below).

Based on the block diagram representation of simple regulation shown in **Figure 3**, we can now decompose any complex gene network using four interconnection topologies: cascade forms, parallel forms, feedback loops, and feedforward loops. These topologies are often intermingled with one another as shown in later examples. In this section, we describe the cascade and parallel forms. **Figure 4(a)** shows an example of cascaded simple regulation blocks. The first output is the product of *F(s)* and *G_Y_(s)*. It is also the input for the second simple regulation. The second or final output is the sum of F(s)G_Y_(s) and the basal production rate F_0_/s multiplied by F(s) and G_Z_(s). This cascade form can be simplified using an equivalent transfer function *G(s)* as shown at the bottom of the **Figure 4(a)**. **Figure 4(b)** shows an example of parallel simple regulations. In this case, the equivalent transfer function *G*(*s*) is the algebraic sum of *G_Y_*(*s*) and *G_Z_*(*s*). Using these simplification methods, any large complex block diagram can be reduced into a single transfer function.

#### 3. Feedback loops

**Figure 5:**
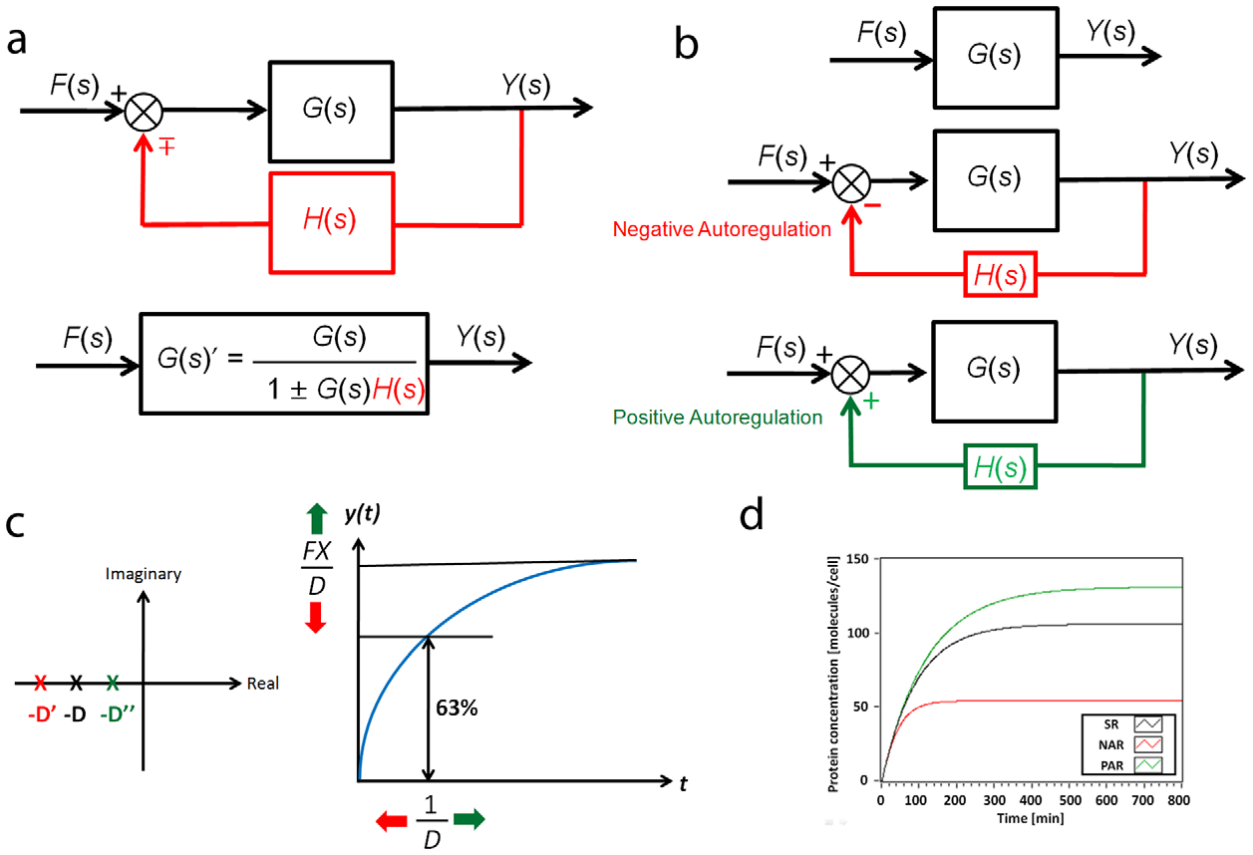
Feedback loops. (a) Block diagram of a feedback loop and its simplified equivalent form. (b) Block diagram of negative autoregulation (NAR) and positive autoregulation (PAR). (c) The effect of H on the stability and behavior. NAR speeds up the response time of the gene expression and decreases the steady state value. In contrast, PAR increases both the response time and steady state value. (d) Simulation results. SR stands for simple regulation. The effects of NAR and PAR on the response time and steady state value are shown.

**Figure 5(a)** shows the block diagram of a feedback loop. Compared to **Figure 3**, there is an additional feedback element *H(s)*. The block diagram can be expressed in the Laplace domain as:
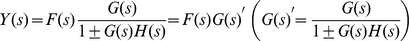
(24)where *G(s)′* is the simplified equivalent transfer function.

#### 4. Case Study I: Autoregulations

Autoregulation is the simplest form of feedback loops and consists of one simple regulation and one feedback loop (**Figure 5(b)**). In simple regulation, x*(*t*) is the only transcription factor that determines the production of *y(t)*. On the other hand, in autoregulation, both x*(t) and y*(t), the active form of *y(t)*, can affect the *y(t)* production. The *y(t)* production function due to y*(t) or *f_y_(t)* requires two Hill functions as in the case of *y(t)* production function due to x*(t). Assuming that the signal *s_y_* that controls the y*(t) formation is an activator:
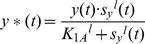
(25)The production function *F_y_*(*t*) with respect to y*(t) can be expressed as:
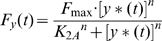
(26)Applying the linearization scheme used in (9–12), we can rewrite (26) as:
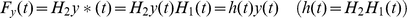
(27)
*h(t)* in (27) is equivalent to *f(t)* in (12). As in (18), if we assume that the signal *s_y_*(*t*) is constant then we can express *H_1_*(*t*) as a constant value *H_1_* and, consequently, *h(t) as H* (*H* = *H_1_ H_2_*).

Negative autoregulation (NAR) occurs when a transcription factor represses the transcription of its own gene (negative feedback). It has been demonstrated that NAR speeds up the response time of the gene expression, decreases the steady state value, and reduces cell-cell variation in protein levels [13], [46]. Using (20) and (24), the transfer function G′(s) can be derived as:
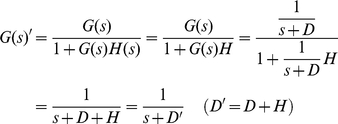
(28)Note that we have a new degradation/dilution constant *D′*. Since both *D* and *H* have positive values in biological systems, *D′* is also positive, indicating that the system is stable based on the pole-zero analysis described previously (**Figure 5(c)**). Furthermore, the time constant of the response (*1/D′*) is greater than *1/D* since *H* is positive. This explains the experimental observation of the decrease of the response time using NAR (**Figure 5(c)** and **(d)**).

Positive autoregulation (PAR) occurs when a transcription factor controls its own protein production rate. In contrast to NAR, the response time is extended and the steady state value and cell-cell variation are increased [13]. Using (20) and (24), the transfer function of PAR is:
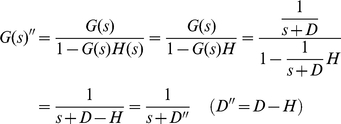
(29)Equation 29 illustrates that, compared to simple regulation, the time constant (*1/D″*) is increased because *D*″ is equal to *D* subtracted by *H*, a positive number. This explains the increase of the response time (**Figure 5(c)** and **(d)**). Additionally, for the system to be stable, – *D″* must be negative (or *H* must be smaller than *D*). In other words, if the positive feedback is “too strong” (*H* is greater than *D*) then the system may become unstable.

#### 5. Case Study II: Negative feedback loop involving two cascaded simple regulations

**Figure 6:**
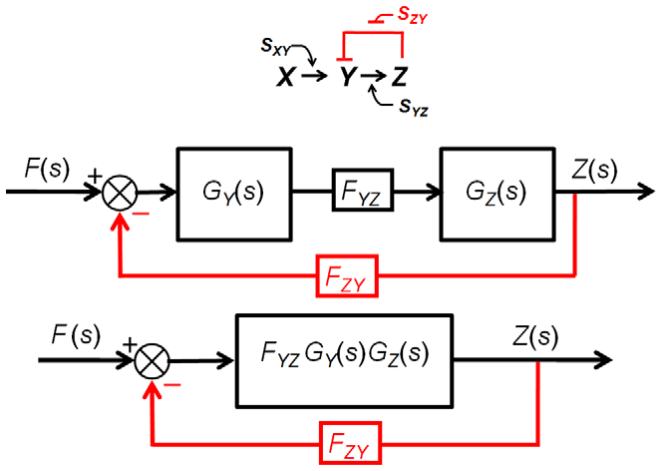
Negative feedback loop involving two cascaded simple regulations. The top block diagram is reduced (simplified) into an equivalent block diagram shown at the bottom. Note that the transfer function in the reduced form is the product of the transfer functions *G_Y_*(s) and *G_Z_*(s) multiplied by *F_YZ_*.

**Figure 7:**
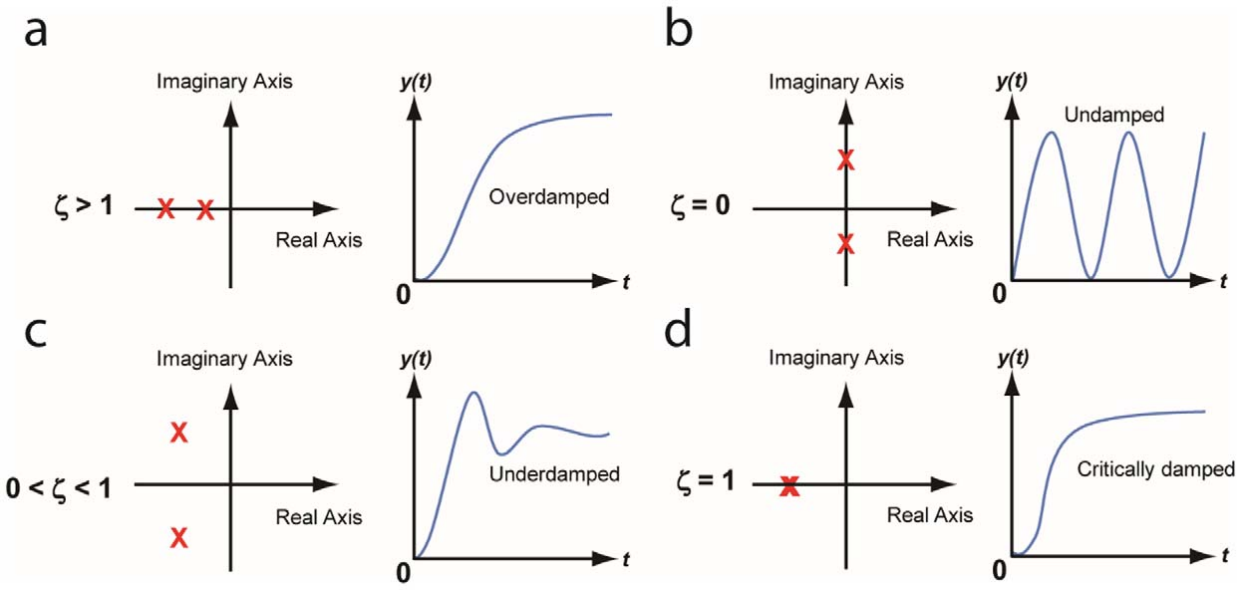
Step response of a second-order system with respect to the damping ratio ζ (the poles are shown as X). (a) Overdamped oscillation. The damping ratio is greater than 1 and the poles are both negative real numbers. The system reaches its steady state without oscillation. As the damping ratio increases, it reaches the steady state slower. (b) Undamped oscillation. Note that all the poles are on the imaginary axis. The damping ratio is zero and there is an oscillation without damping. (c) Underdamped oscillation. The damping ratio is between 0 and 1, and the poles are complex numbers with the negative real part. The oscillation gradually decreases to zero as the system reaches its steady state. (d) Critically damped oscillation. The steady state is reached in the fastest way without oscillation. The two poles have the same negative value [42].

**Figure 6** shows the block diagram of a negative feedback loop that consists of two cascaded simple regulations. *X_gene_* activates *Y_gene_* in the presence of signal *S_XY_*. *Y_gene_* then activates *Z_gene_* in the presence of *S_YZ_*, and *Z_gene_* at the same time represses *Y_gene_* in the absence of *S_ZY_*. If we assume that the basal protein production rate is negligible (zero) the transfer function becomes second-order system and there are well-established linear control theory tools for analyzing second-order systems. However, as we will show in subsequent section, the results can be extended to cases where the basal rates are not zero. Using (24), the transfer function can be expressed as:
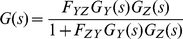
(30)Substituting *G_Y_(s)* and *G_Z_(s) by* 1/(*s+D_Y_*) and 1/(*s+D_Z_*), (30) becomes:
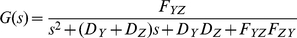
(31)Now we have a second-order transfer function *G(s)*. Whereas varying the first-order transfer function parameter *D* changes only the response time (1/*D*), changing the parameters (*F_YZ_*, *F_ZY_*, *D_y_* and *D_z_*) of a second-order transfer function can influence both the speed and form of the system response [42]. *G*(*s*) can be restated in a general second-order form:
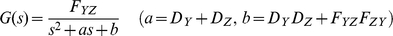
(32)The natural frequency *ω_n_* of a second-order system is defined as the frequency of an undamped oscillation [42].
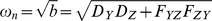
(33)Without damping, the poles would be on the imaginary axis as shown in **Figure 7(b)**. Equation (33) shows that the natural frequency can be tuned by varying the strength of both *F_YZ_ and F_ZY_*.

For a damped system, the damping ratio *ζ* is defined as [42]:
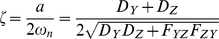
(34)Various step responses of a second-order system with respect to the damping ratio *ζ* are shown in **Figure 7**. The damped natural frequency *ω_n_* is defined as [42]:
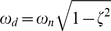
(35)The poles of an undamped oscillating system lie on the imaginary axis, and thus the system is said to be marginally stable [42]. Also, note that all the poles in **Figure 7** lie on the left half of the plane except for the ones that belong to an undamped oscillation. For an underdamped oscillation, additional specifications can be defined and used to predict the response in detail, including the peak time, percent overshoot, settling time, and rise time. Using a numerical approach, rise time can be computed even though there is no precise analytical solution [42]. All these specifications can be expressed in terms of *F_YZ_*, *F_ZY_*, *D_Y_* and *D_Z_*.

#### 6. Case Study III: Tunable synthetic gene oscillators

Building tunable synthetic gene oscillators has been a central area of focus for systems and synthetic biologists [47]–[49]. An oscillating system is basically an undamped second-order transfer function (**Figure 7(b)**). For a system to have no damping, *a* (being equal to *D_Y_* + *D_Z_*) in (34) must be zero. Therefore, an additional negative term is required to decrease the value of *a* to zero (or close to zero from a practical point of view) since the degradation/dilution constants *D_Y_* and *D_Z_* are positive numbers. Using the transfer function method, we will show that the use of positive autoregulation can lead to undamped oscillations and that by varying the strength of positive autoregulation we can tune the oscillation frequency.

**Figure 8:**
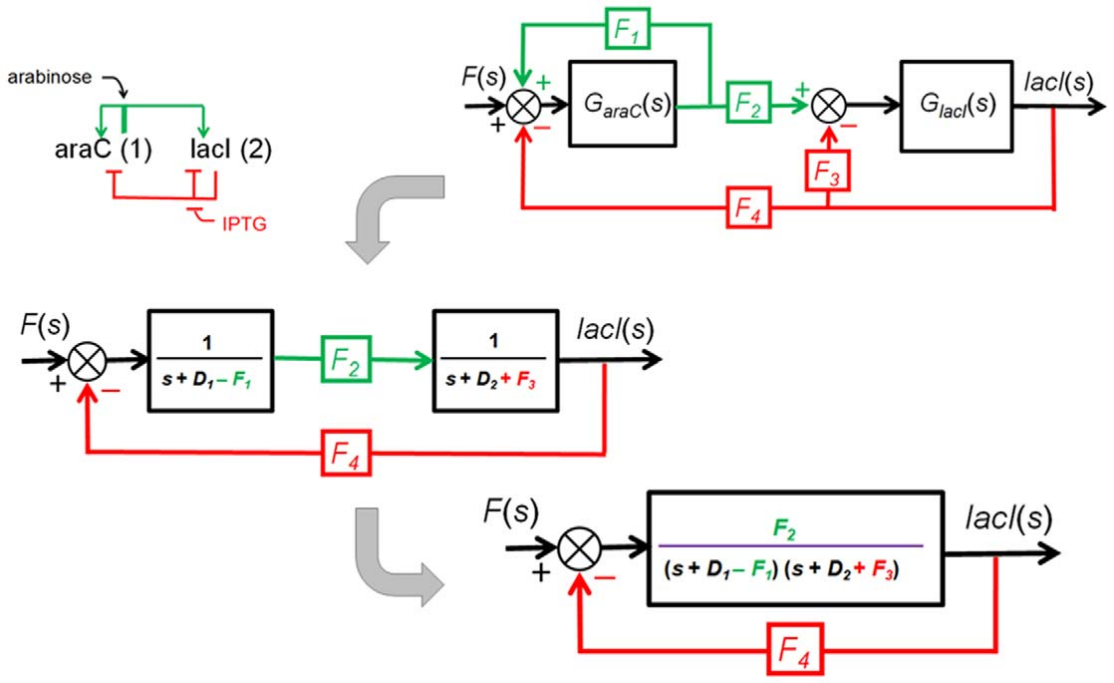
Tunable synthetic gene oscillator. The top block diagram is reduced into the block diagram in the middle by removing positive and negative autoregulation. Then, the two cascaded transfer functions are reduced into a single equivalent transfer function, as shown in the bottom block diagram.

A tunable synthetic gene oscillator is shown in **Figure 8** [47]. The genes araC (denoted as 1) and lacI (denoted as 2) have identical hybrid promoters that can be activated by AraC in the presence of arabinose and repressed by LacI in the absence of IPTG. As shown in the figure, araC has a positive autoregulation while LacI a negative autoregulation. *F_1_* and *F_3_* are determined by the signals, arabinose and IPTG, respectively. The uppermost block diagram in **Figure 8** can be simplified by removing autoregulations (28 and 29). It can be further simplified since it has two cascaded elements. The final equivalent block diagram with a single transfer function is shown at the bottom of the figure. Using (24), the overall transfer function can be expressed as:
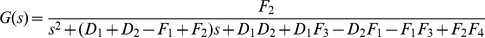
(36)According to (36), *a* ( = *D_1_* + *D_2_* − *F_1_* + *F_2_*) must be zero (or close to zero) for an undamped oscillation. It is clear that *F_1_*, the only negative term created by the positive autoregulation of *araC*, can contribute to decreasing *a* to zero. This explains why positive autoregulation is needed for an undamped oscillation in biological systems. The natural frequency of the response is:
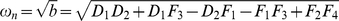
(37)


Equation 37 shows that both the parameters influenced by arabinose (*F_1_* and *F_2_*) and parameters influenced by IPTG (*F_3_* and *F_4_*) can change or tune the oscillating frequency. Since *F_1_* and *F_2_* in (37) contribute to both positive and negative terms, it is difficult to predict their effect on the frequency. Likewise, *F_3_* and *F_4_* are also found in both positive and negative terms in (37), again making the prediction difficult. Experimentally, the IPTG concentration and oscillating frequency show a non-monotonic relationship [47].

Using the transfer function method, we can identify a way of changing such a non-monotonic relationship into a monotonic one. For example, if the negative autoregulation for *lacl* is removed from the network (by removing the repression operator site from the promoter), *D_1_F_3_* and −*F_1_F_3_* are eliminated from (37). The natural frequency then becomes:
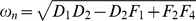
(38)Now, *F_4_* belongs to a positive term, meaning that as the IPTG concentration is raised the oscillating frequency also increases, and vice versa, indicating that IPTG can now cause a monotonic behavior.

In order for an undamped system (*a* = 0) to be marginally stable, *b* in (37) must be greater than zero so that the two poles lie separately on the imaginary axis [42]:
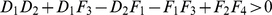
(39)There are two negative terms (– *D_2_F_1_* and – *F_1_F_3_*) that can make the system unstable and they are related to both arabinose (*F_1_*) and IPTG (*F_3_*). However, since *F_1_* is present in both terms and not in any of the positive terms, decreasing the arabinose concentration (and not the IPTG concentration) seems to be an effective solution when there is an issue of instability.

#### 7. Case Study IV: Eliminating the effect of the basal production rate

**Figure 9:**
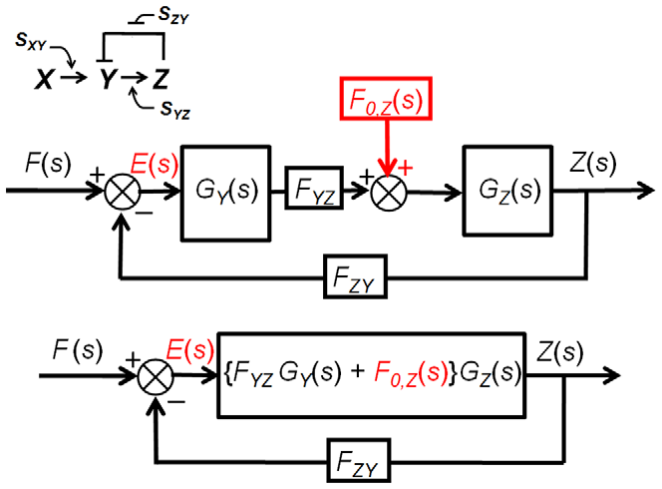
Negative feedback loop involving two cascaded simple regulations with the Laplace transform of the basal production rate *F_0,Z_*(*s*) added. The top block diagram is reduced (simplified) into an equivalent block diagram shown at the bottom.

In this section, we will demonstrate how the effect of the basal production rate *f_0_*(*t*) can be removed using a second-order negative feedback system described earlier. If we add the Laplace transform of the basal *Z* protein production rate *F_0,Z_*(*s*) to the model shown in the Figure 6, as illustrated in Figure 9, the transform of the output *Z*(*s*) is given by:
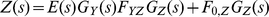
(40)Note that *E*(*s*) is the error (difference) between F(s) and *F_ZY_Z*(*s*). It is analogous to the concept of steady-state error in the presence of a disturbance (*F_0,Z_*(*s*) in our case) [42]. Substituting 
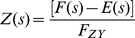
 into (40) and solving for *E*(*s*), we get:
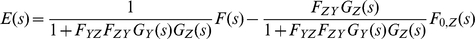
(41)where the first term can be regarded as a transfer function relating *E*(*s*) to *F*(*s*) and the second term relating *E*(*s*) to *F_0,Z_*(*s*).

Applying the final value theorem to Eq. (41), we obtain:
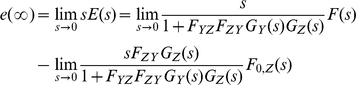
(42)As we assume the basal rate is constant *F_0,Z_*, *F_0,Z_*(*s*) becomes *F_0,Z_*/s. Substituting this value into the second term of (42), the steady-state error component due to the basal rate can be found as:
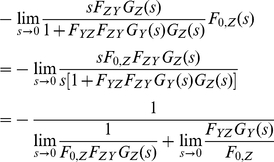
(43)Equation (43) can provide important insight about reducing the steady-state error due to basal activity by tuning the strength of *F_YZ_* or *F_ZY_*.

#### 8. Case Study V: A synthetic oscillator involving four cascaded simple regulation loops

A second order system with a negative feedback loop described earlier could be efficiently analyzed using a pole/zero plot and various formulas. Even though we do not have such formulas for higher order systems, pole/zero plots can still be useful for predicting their behavior.

**Figure 10:**
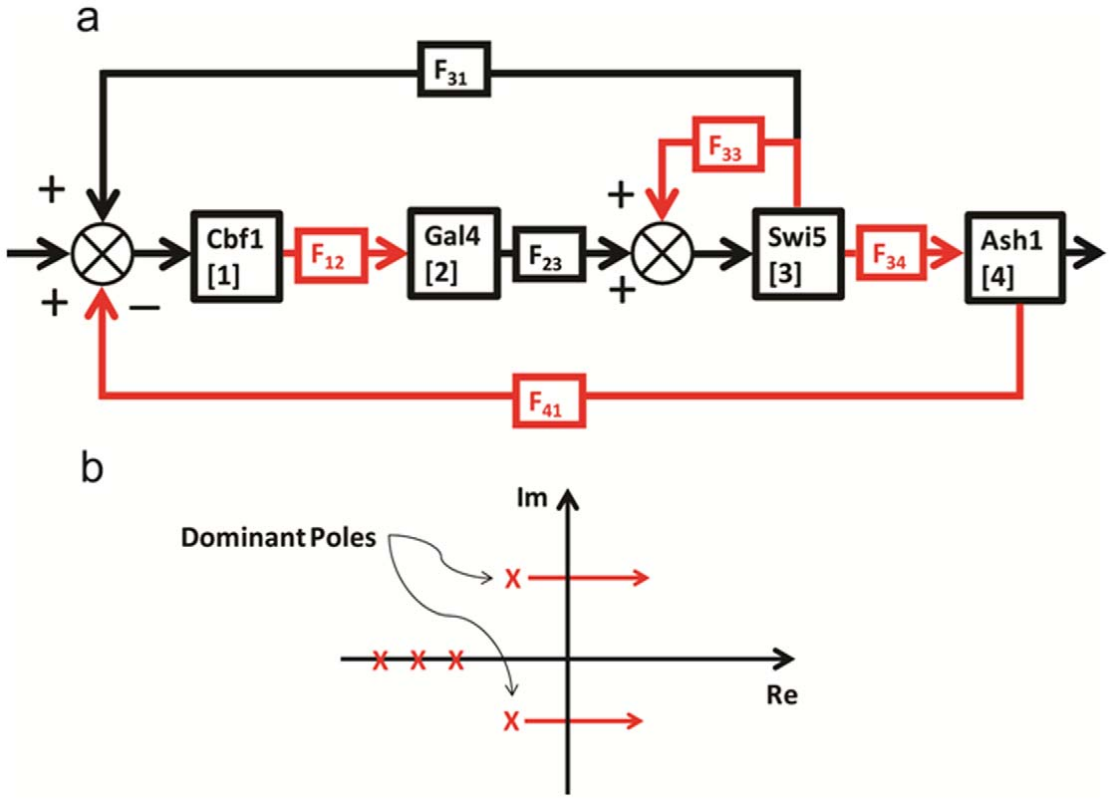
Negative feedback loop involving four-cascaded simple. (a) Increasing the values of F_12_, F_33_, F_34_, and F_41_ contributes to generating an oscillatory behavior (shown in red). (b) Increasing the values of F_12_, F_33_, F_34_, and F_41_ causes the dominant poles to cross the imaginary axis. When the dominant poles are exactly on the axis, the system exhibits an undamped oscillation.

**Figure 10** shows the block diagram of a fourth order system with various feedbacks. It is a synthetic network called IRMA (*In vivo* Reverse-engineering and Modeling techniques Assessment) that consists of four genes (*CBF1*, *GAL4*, *SWI5*, and *ASH1*) in *Saccharomyces Cerevisiae* [50]. It has been demonstrated both computationally (using a non-linear model) and experimentally that the network could be turned into an oscillator by changing various parameters (Michaelis-Menten coefficient, Hill coefficient, etc) that control the strength of the interactions among genes.

An equivalent model was built using the transfer function method, as shown in **Figure 10(a)**, where the strength of the interactions was determined by changing the values of the constants (F_12_, F_23_, F_31_, F_33_, F_34_, and F_41_) shown in the figure. The numbers 1, 2, 3, and 4 in subscript represent the four genes mentioned earlier, respectively. As described in the paper [50], we were able to demonstrate that increasing the values of F_12_, F_33_, F_34_, and F_41_ contributes to generating an oscillatory behavior. **Figure 10(b)** shows that as we increase the values of those constants, the dominant poles (two poles that have the least real values) cross the imaginary axis. When the dominant poles are exactly on the axis, the system exhibits an undamped oscillation, similar to the behavior of a second order system shown in **Figure 7** (see Movie S1 and File S1).

#### 9. Case Study VI: Interconnected feedforward loops

**Figure 11:**
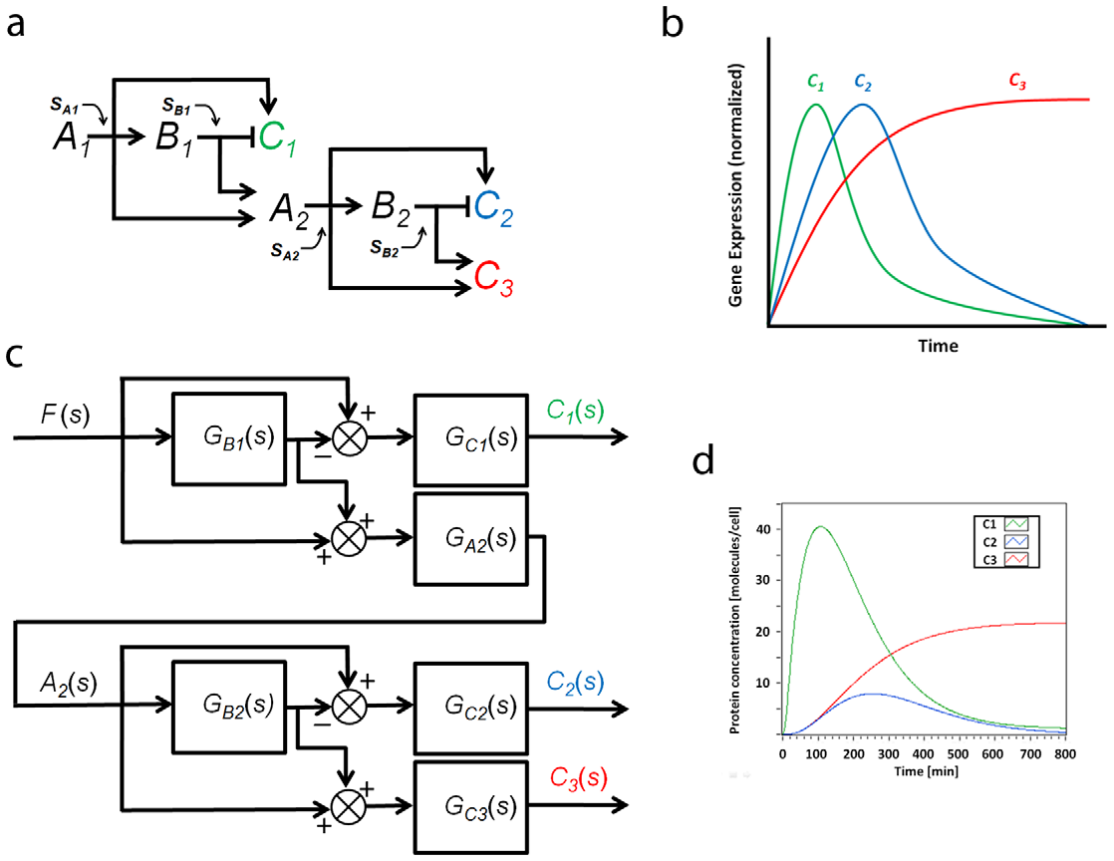
Interconnected feed forward loops. (**a**) **A simplified schematic diagram.** Two sets of C1-FFL and Ic1-FFL in parallel are connected in cascade. (**b**) The sequential expression of C1, C2, and C3. Ic1-FFLs generate pulses of C1 and C2, and C1-FFLs create delays in C2 and C3 expression. (**c**) Block diagram. The protein production constants “F”s are not shown in the diagram. (**d**) Simulation result. The sequential expression of the three genes is shown. Note that if the values of the protein concentrations are normalized, the expression pattern will be equivalent to the one shown in (b).

Feedforward loops (FFL) are network motifs [13] that combine into more complex and larger networks (e.g. in *Bacillus Subtilis* [51]). The coherent type-1 feedforward loop (C1-FFL) and incoherent type-1 feedforward loop (Ic1-FFL) are the most abundant FFL types [52]. In this section, we will describe how transfer function method can be used to model a network that consists of interconnected feedforward loops. **Figure 11(a)** shows a simplified schematic diagram of the network. Two sets of C1-FFL and Ic1-FFL in parallel are connected in cascade. Ic1-FFLs generate pulses of C1 and C2, and C1-FFLs create delays in C2 and C3 expression. **Figure 11(b)** shows a schematic diagram that illustrates the sequential expression of C1, C2, and C3. **Figure 11(c)** shows the block diagram of the network. Using the transfer function method, the sequential expression of the three genes can be simulated as shown in **Figure 11(d)**. Note that if the values of the protein concentrations are normalized, the expression pattern will be equivalent to the one shown in **Figure 11(b)**.

### B. Linear State-Space Method for Gene Network Modeling

A state is a complete summary of the status of a system at a particular point in time, and it is described by the values of a set of state variables [53]. Based on the linearization scheme presented previously, a linear state-space method can be applied to model gene networks. There are many benefits in using the linear state-space approach. First, it allows time-varying systems, meaning that the models can include time-varying signals. This was not possible in the case of transfer function-based models described earlier. Secondly, large complex networks that are multiple-input multiple-output (MIMO) systems can be represented in a compact way, using vectors and matrices. Thirdly, the dynamic behavior of a system can be understood using the eigenvalues and eigenvectors. The eigenvalues are equivalent to the poles discussed in the transfer function method. Finally, we can build a stochastic linear state-space model that enables us to utilize a spectrum of tools available for optimal/robust estimation/control for gene network modeling [35]–[37]. As an example, we illustrate that the Kalman filter, one of the well-established optimal estimation algorithms in science and engineering, can be applied towards modeling a simple two-gene network.

In the linear model of simple regulation (linear ODE form shown in (13)), the system involves two proteins, *x*(*t*) and *y*(*t*), with units of concentration per cell. If we consider gene *y* as our system of interest, then and the state vector **s** can be expressed as:
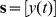
(44)The state-space model can be represented as [53]:
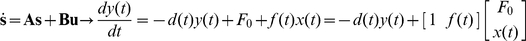


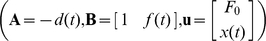
(45)where ***u*** is the input vector. Note that we do not have to assume that *f*(*t*) and *d*(*t*) are time-invariant as in the transfer function case (18).

**Figure 12:**
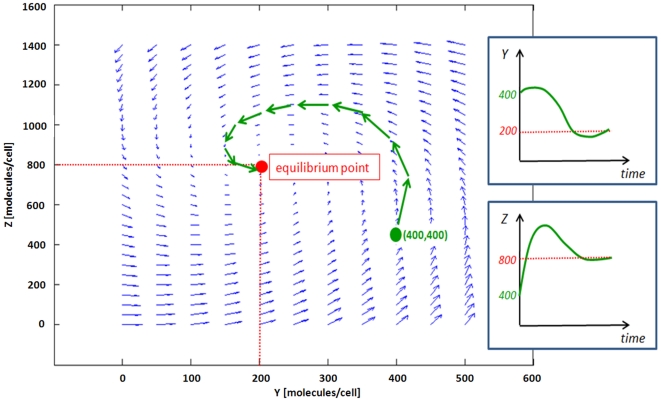
Vector field of the negative feedback loop. The direction and magnitude of each vector is determined by (46). When the initial value is (400,400), the trajectory eventually converges to the equilibrium point (200,800) (green arrows). Damped oscillations of both *Y* and *Z* expressions are also shown in small boxes.

The state-space method is analogous to the transfer function method in many ways. Therefore, in this section we present only the case of a negative feedback loop involving two cascaded simple regulations. The network shown in **Figure 6** can be written as a state-space model:
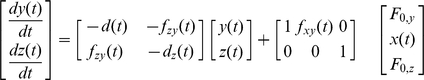


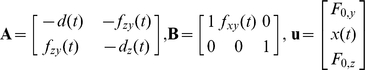
(46)where *f_ab_*(*t*) denotes a function (shown in (12)) related to the production of protein *b* by protein *a*. *F_0,a_* represents the basal production rate of protein *a*. We can predict the behavior and stability of the system using the eigenvalues and eigenvectors of the matrix ***A*** [53], [54]. For example, assuming *d_y_*(*t*) = *d_z_*(*t*) = 0.01 (constant), *f_zy_*(*t*) = 0.01 (constant), *f_yz_*(*t*) = 0.04 (constant), *f_xy_*(*t*)·*x*(*t*) = 10 (constant), the eigenvalues (*λ_1_* and *λ_2_*) and eigenvectors (***φ*** *_1_* and ***φ*** *_2_*) of ***A*** can be calculated as:
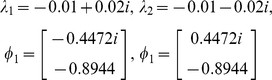
(47)Since the eigenvalues are equivalent to the poles, we can immediately predict that *y* and *z* will exhibit underdamped oscillations and the system is stable. The solution can be written as:
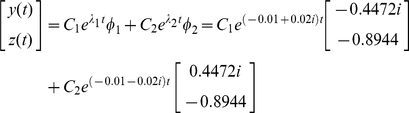
(48)where *C*_1_ and *C*_2_ are determined by the initial values of *y* and *z*. Depending on the initial values, the time-dependent values of *y* and *z* can follow an infinite number of paths as shown in the vector field (**Figure 12**). However, regardless of the initial values, the figure shows that all the trajectories will eventually converge into an equilibrium point. This equilibrium point can also be computed by solving *dy*(*t*)/*dt* = 0 and *dz*(*t*)/*dt* = 0 in (46). Note that the damped oscillatory behaviors that were predicted using the eigenvalues are observed in both *Y* and *Z* expressions (**Figure 12**).

#### 1. Case Study VII: Optimal estimation in gene network measurements

Stochastic optimal estimation [18], [19], [43], [45] and control methods aim to determine the best strategy for estimating or controlling a dynamic system in the presence of uncertainty [35]. This objective is common to many fields, including engineering, science, and economics [35]. Assume that, in the case of simple regulation x→y, we are given noisy time-series fluorescence measurement data of *y* protein [55]–[57]. Would it be possible to “optimally” estimate protein *x* which is also fluctuating due to noise? In this section, we will illustrate that the Kalman filter, a well-established optimal estimation algorithm, can be applied for stochastic modeling of a simple regulation (two-gene network). Before we discuss the stochastic model of simple regulation, it is important to note that there are two different noise contributions in gene networks [18], [58], [59]. “Intrinsic noise” is generated by the inherent stochasticity of biochemical processes, such as transcription and translation, which are directly related to the expression of a specific gene. On the other hand, the environment including other cellular components (mitochondria, microtubules, etc) that indirectly influence the expression of *y* gene, contribute to the “extrinsic noise”.

First, the least squares estimation is used to estimate the constant concentration of protein *x* (without noise), given noisy measurement data of protein *y*. Using the Euler's method, equation (13) can be expressed as:
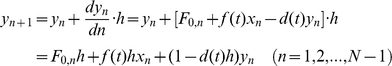
(49)where *h* is the step size. For simplicity, we will assume *f*(*t*) and *d*(*t*) are constant (*F* and *D*) and the basal protein production rate and initial value of *y* are zero (*F*_0,n_ = *y*_1_ = 0):
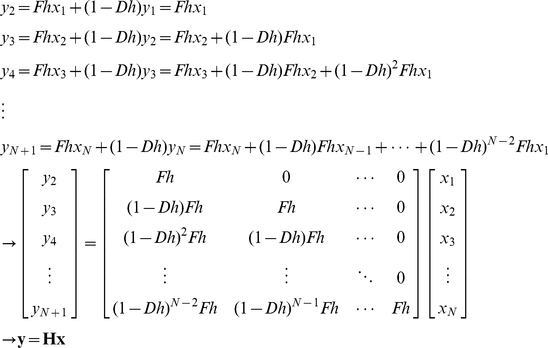
(50)which can be compactly shown as ***y*** = ***Hx***. Since the measurement data vector ***y*** has noise:
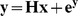
(51)where ***e*** *^y^* is assumed to be a Gaussian zero-mean white noise vector. **Σ**, the covariance matrix of ***e*** *^y^*, can be shown as:
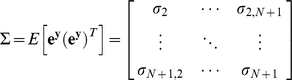
(52)Note that in this formulation protein x is noise-free and thus has no stochastic influence on y. Using the least squares method, the estimated ***x*** can be calculated as:
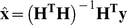
(53)Now, we will use the Kalman filter for estimating noisy ***x*** from noisy ***y*** (Methods: Derivation of the discrete-time Kalman filter for simple regulation). For estimating the concentration of ***x*** with a noise vector ***e*** *^x^* (intrinsic and extrinsic noise combined), following equations can be used:
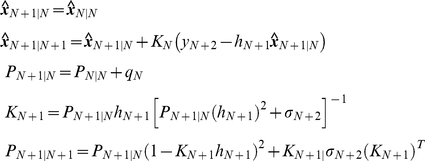
(54)


**Figure 13:**
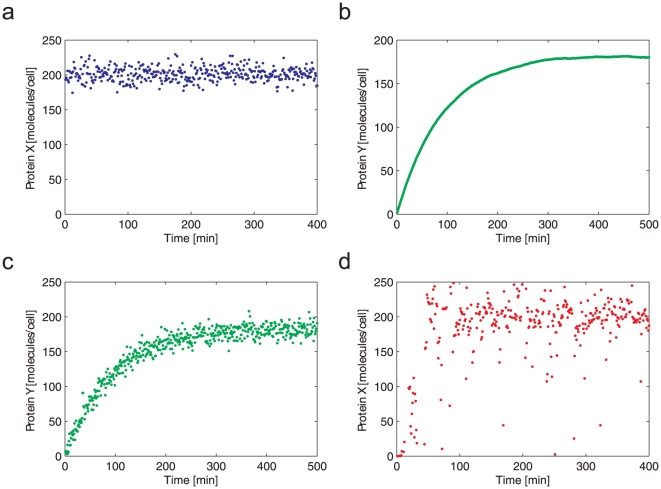
Estimation of *x* using the Kalman filter. (a) ***x*** is the sum of a constant value (200 molecules/cell) vector and Gaussian zero-mean white noise vector with a standard deviation of 10. It is assumed to have both intrinsic and extrinsic noise components. (b) ***y*** produced by the action of x illustrated in panel a. (c) Extrinsic noise ***e*** ^y^ is added to ***y*** shown in panel b. (d) The estimated ***x*** using the Kalman filter is shown.

**Figure 13(a)** shows ***x***, which is the sum of a constant value (200 molecules/cell) vector and Gaussian zero-mean white noise vector with a standard deviation of 10. The noise is assumed to have both intrinsic and extrinsic components. ***y*** produced by the action of ***x*** is shown in **Figure 13(b)**. Note that the noise has been reduced significantly. The noise in **Figure 13(b)** can be regarded to be as intrinsic only, indicating that a simple regulation can behave as a low-pass filter (removes high-frequency signals which are noise components in this example) [32]. In **Figure 13(c)**, extrinsic noise ***e*** *^y^* described in (50) is added to ***y***, and the total noise is composed of both intrinsic and extrinsic components. For the simulation, we assume that the extrinsic noise ***e*** *^y^* is a Gaussian zero-mean white noise vector with the standard deviation 9. **Figure 13(d)** shows the estimated ***x*** using the Kalman filter, given ***y*** in **Figure 13(c)**. Even though it does not exactly duplicate ***x*** shown in (a), it is the “optimal” estimation of ***x***.

#### 2. Case Study VIII: Analysis of a six-node gene network

**Figure 14:**
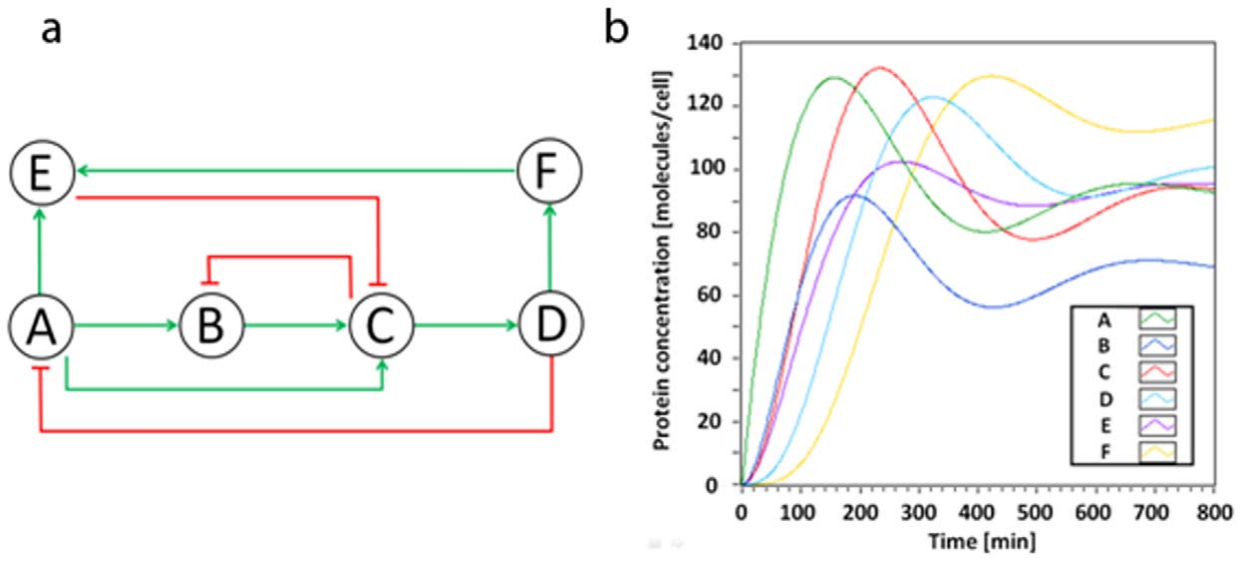
A six-node gene network. (a) A schematic illustration. (b) Simulation result based on the linear state-space method.

Here, we will illustrate the utility of the state-space method for the analysis of a 6-node network (**Figure 14(a)**). There are a few dynamic features that we may intuitively extract from the figure based on our previous discussions. For example, there is a coherent type-1 feedforward loop (C1-FFL) embedded in the network (that consists of A, B, and C), which suggests that gene C is expressed after the expression of gene B (there is a delay in the C expression).

The 6-node gene network can be modeled using the following state-space formulation:
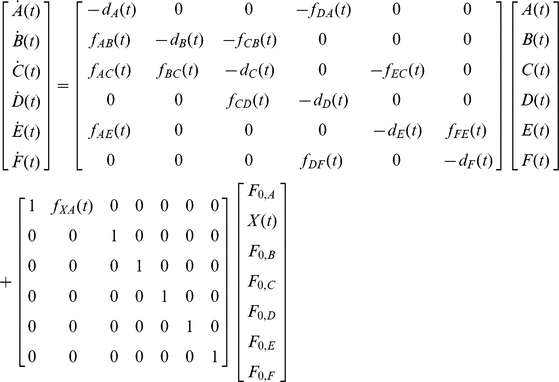
(55)where *f_AB_*(*t*) is analogous to *f*(*t*) in (13) and accounts for the relationship between *A*(*t*) (transcription factor) to *B*(*t*) (expressed protein). It is assumed that gene A is expressed by an arbitrary gene *X*, which is shown as an input in the model. **Figure 14(b)** illustrates that intuitive and qualitative information about the dynamic behavior can be acquired using the linear state-space method. The delay in gene *C* expression compared to the expression of gene *B* is also shown.

### C. Further Discussion and Conclusion

We demonstrated that well-established tools of linear control theory can be used to model gene networks and explain experimental observations in a highly intuitive way. Methods such as the transfer function (frequency domain) and linear state-space (time domain) were applied to reveal inherent characteristics and predict the dynamic behavior of various gene network topologies, including cascade/parallel forms, feedback loops, and feedforward loops. Additionally, we showed that well-established optimal estimation tools, such as the Kalman filter, can be used in the context of gene network modeling in the presence of noise.

While we assumed that multiple transcription factors act on a single gene in an additive/subtractive way, in biological systems positive or negative cooperativity, and mutual exclusion can also be observed. Combinational logic is one approach that can be used for determining the net effect of multiple inputs at such junctions [13], [30]. We expect that hybrid models that combine both continuous and logical approaches may provide additional insight.

## Methods

### A. Simulations

For simulations, the value of degradation/dilution constant (D) was 0.01/min and *f*(*t*) was set as a constant (*F*) that ranged from 0.01 to 0.1/min. The input *x*(*t*) had a fixed value between 100 and 1000 molecules/cell and the typical basal protein production rate was set as 0.1 molecules/min.

### B. Derivation of the discrete-time Kalman filter for simple regulation

#### 1. Recursive least squares estimation

For the estimation of ***x***, a new piece of information y_new_ can be used without repeating calculations already done on **y_old_** through the recursive least squares estimation [34], [36]. With a new piece of updated information, (51) can be expressed as:
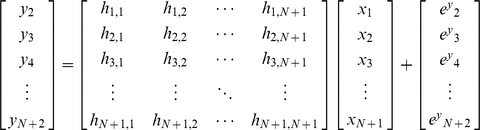
(56)From (56), it can be stated that:
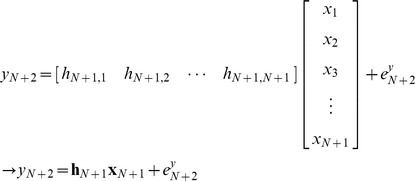
(57)A linear recursive estimator can be written as [36]:
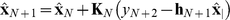
(58)where ***K***_N_ is a gain matrix.

The mean of the estimation error 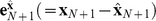 can be computed as [36]:
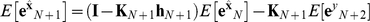
(59)The covariance matrix ***P*** of the estimation error 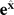 is:
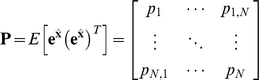
(60)A recursive formula for the calculation of the estimation-error covariance also can be shown as [36]:
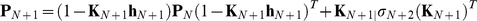
(61)The gain matrix can be expressed as [36]:
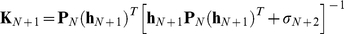
(62)


#### 2. Propagation of the mean and covariance

Suppose we have the following linear discrete-time system [36]:
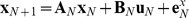
(63)where 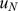 is a known input and 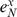 is Gaussian zero-mean white noise. The covariance matrix of ***e*** ^x^ can be shown as:
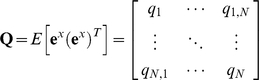
(64)If we take the expected value of both sides of (63):
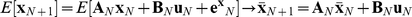
(65)which shows the propagation of the mean of ***x*** *_N_* [36]. The propagation of the covariance can be shown as [36]:
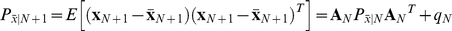
(66)


#### 3. Derivation of the discrete-time Kalman filter

If we have all of the measurements up to and including time N+1 available for our estimate of 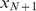, then we can form *a posteriori* estimate denoted as 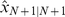. If we have all of the measurements before (not including) time N+1 available for our estimate of 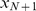, then we can form *a priori* estimate denoted as 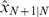
_._ Similarly, 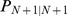 denotes the covariance of 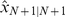 and 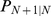 denotes the covariance of 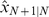. Assuming that the initial state 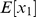 is given:
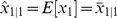
(67)Using (65) that describes the propagation of the mean of 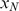, we obtain:
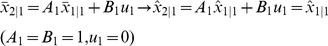
(68)The reasoning can be extended as following:
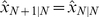
(69)Assuming 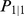, the covariance of the initial estimate 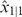, is given and using (66):
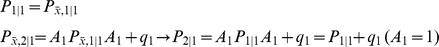
(70)The reasoning can be extended as following:
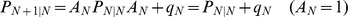
(71)Now we have the time-update equations for 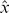 and *P* and we need the measurement-update equations for 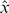 and *P*. Utilizing the recursive least squares development (56–62), the discrete-time Kalman filter for simple regulation can be summarized as:
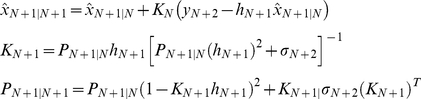
(72)


## Supporting Information

File S1 LabVIEW VI file for Case Study V.
(0.06 MB TXT)

Movie S1 Movie file for Case Study V.
(1.16 MB MOV)
